# Complete chloroplast genome of *Ulva pertusa*, one of the causal species of green macroalgal blooms in the coastal waters of Qinhuangdao, China

**DOI:** 10.1080/23802359.2020.1723448

**Published:** 2020-02-06

**Authors:** Han Hongbin, Yan Li, Song Wei, Wang Zongling, Zhang Xuelei

**Affiliations:** a First Institute of Oceanography, the Ministry of Natural Resources, Qingdao, China;; b Laboratory of Marine Ecology and Environmental Science, Pilot National Laboratory for Marine Science and Technology (Qingdao), Qingdao, China;; c Laboratory of Marine Ecology and Environmental Science, Qingdao National Laboratory for Marine Science and Technology, Qingdao, China

**Keywords:** Macroalgae blooms, chloroplast genome, *Ulva pertusa*, phylogenetic analysis, *Ulva fasciata*

## Abstract

Since 2015, macroalgae blooms have appeared along the Qinhuangdao coast of the Bohai Sea in China and they have recurred annually during the months of April to September. One of the causal species that results in the macroalgal blooms, *Ulva pertusa*, has been detrimental to the environment and ecosystem along the coast of the Qinhuangdao, China. In the present study, we sequenced the chloroplast genome of *U. pertusa* for the first time (GenBank accession number MN853875) and found that the annular genome comprised 104,380 base pairs, including 71 protein-coding genes, 26 tRNAs, and 2 rRNAs. We then constructed a phylogenetic tree of *U. pertusa* and 17 other species based on core genes, which showed that *U. pertusa* is the closest sister species of *U. fasciata.*

Since 2007, the world’s largest macroalgae blooms consisted primarily of *Ulva prolifera* and recurred on the coast of Qingdao City in Shandong Province of China, which has adversely affected the local environment and tourism (Liu et al. 2009, 2010, 2013; Zhao et al. 2013; Zhang et al. 2017). However, in 2015, a novel macroalgae bloom occurred in the coastal waters of Qinhuangdao City on the western coast of the Bohai Sea making it the second coastal area in China influenced by macroalgae blooms (Han et al. 2019; Song, Han, et al. 2019; Song, Wang, et al. 2019). The bloom’s process had obvious species succession exhibited by three distinct development stages (Song, Han, et al. 2019; Song, Wang, et al. 2019). *Ulva pertusa* was the dominant species of stage I, which occurred from late April to mid-May (Song, Wang, et al. 2019). Compared with the mitogenome, the chloroplast genome contains more protein-coding genes, which plays an important role in cytoplasmic inheritance, plant phylogeny, DNA barcoding, and genetic diversity (Cai et al. 2017, 2018). Therefore, in this study, we determined the complete chloroplast genome sequence of *Ulva pertusa*.

*Ulva pertusa* was collected from the Qinhunangdao coastal area of the Hebei Province (39°49′54.69″N, 119°31′30.50″E). The specimens were preserved at the Marine Ecology Research Center of the First Institute Oceanography, Ministry of Natural Resources in Qingdao (Accession number: KSC06). The shape of genome of *U. pertusa* is annular with GenBank accession number MN853875. The content of A + T is 74.34%. The complete chloroplast genome sequence is 104,380 bps long. There are 71 protein-coding genes in the genome, including 15 *psb* genes, 11 *rps* genes, 10 *rpl* genes, 6 *psa* genes, 6 *atp* genes, 5 *pet* genes, 5 *ycf* genes, and 4 *rpo* genes. In addition, there are some genes that appeared only once in the genome, including *acc*, *ccs*, *cem*, *chl*, *clp*, *fts*, *inf*, *rbcL* and *tufA* genes. Furthermore, there are 26 tRNAs and 2 rRNAs (*rrl* and *rrs*) which are non-coding genes in the genome. All the coding genes begin with ATG except for *rps19*, which begins with AAT. The termination codons for *rpoA*, *rpl2*, *rpl23*, *psbT*, *psaA*, *rpl20*, *chlI* and *atpI* are TAG. In addition, *rpoA* terminates with TGA and the rest of the 63 genes end with TAA.

To investigate the phylogenetic status of *U. pertusa*, a maximum-likelihood (ML) phylogenetic tree including *U. pertusa* and other 30 species was constructed using the software MEGA-X with 1000 bootstrap replicates (Kumar et al. 2018). As shown in the phylogenetic tree (Figure 1), *U. pertusa* was most closely related to *U. fasciata*.

**Figure 1.**
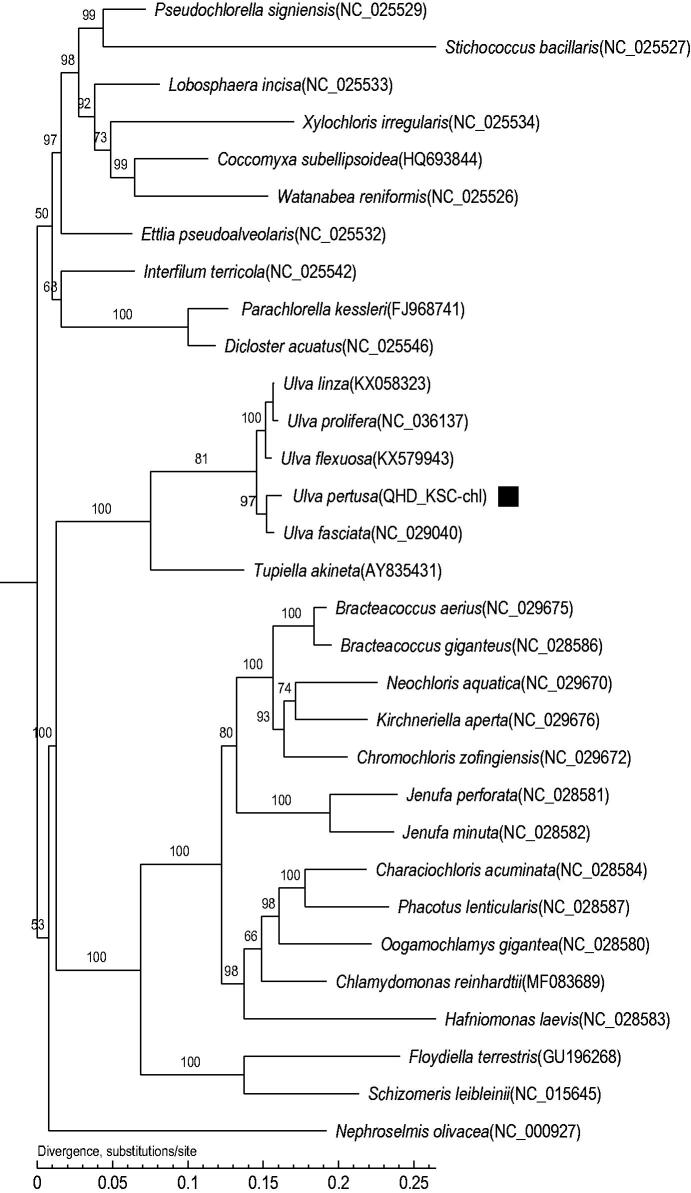
Maximum-likelihood (ML) tree based on the complete chloroplast genome sequences of 31 species. The numbers on the branches are bootstrap values.

In this study, we reported and characterized the complete chloroplast genome sequence of *U. pertusa*, which will be useful for studying genetic diversity and phylogenetic history of *U. pertusa* and its related species.
